# Dietary supplementation with combined extracts from garlic (*Allium sativum*), brown seaweed (*Undaria pinnatifida*), and pinecone (*Pinus koraiensis*) improves milk production in Holstein cows under heat stress conditions

**DOI:** 10.5713/ajas.19.0536

**Published:** 2019-11-12

**Authors:** Jae-Sung Lee, Sukyung Kang, Min-Jeong Kim, Sung-Gu Han, Hong-Gu Lee

**Affiliations:** 1 Department of Animal Science and Technology, Sanghuh College of Life Sciences, Konkuk University, Seoul 05029, Korea; 2 Department of Food Science and Biotechnology of Animal Resources, Sanghuh College of Life Sciences, Konkuk University, Seoul 05029, Korea; 3 Team of An Educational Program for Specialists in Global Animal Science, Brain Korea 21 Plus Project, Sanghuh College of Life Sciences, Konkuk University, Seoul 05029, Korea

**Keywords:** Dairy Cow, Temperature-humidity Index, Heat Stress, Reproductive Trait, Feed Additive

## Abstract

**Objective:**

This study was conducted to examine the effects of a mixture of pinecone oil, garlic, and brown seaweed extracts (PGBE) on milk production traits as well as physiological and ethological parameters in Holstein cows during the summer season (24 May to 03 July 2015, Korea).

**Methods:**

Among the extract combinations tested, we found that the level of 2,2′-azino-bis (3-ethylberzothiazoline-6-sulphonic acid) cation radical scavenging activity of the 0.16% PBGE complex at ratio of 1:1:1 (vol/vol) was comparable to that of the control (ascorbic acid; 1 mg/mL). Additionally, the PBGE complex reduced lipopolysaccharide-induced COX-2 expression in bovine mammary epithelial cells. Based on these findings, 40 lactating Holstein cows were used to measure the effects of PBGE complex at ratio of 1:1:1 (vol/vol) on milk production, immune response, metabolites, and behavior patterns by dividing the cows into two groups fed diets containing PGBE complex (n = 20; 0.016%/kg feed dry matter basis) or not containing PGBE complex (control, n = 20) for 40 d.

**Results:**

Results showed that PGBE complex did not influence milk composition, eating and ear surface temperature patterns, immune response, or metabolic parameters but promoted average milk yield throughout the experimental period. Additionally, a tendency of higher total antioxidant capacity and glutathione in the PGBE group was observed compared to the those in the control. When the temperature-humidity index (THI) exceeded 72 (average THI = 73.8), PGBE complex-fed cows experiencing heat stress showed increased milk yield and a tendency of increased rumination compared to the control.

**Conclusion:**

We suggest that incorporation of a combined mixture of 0.016% PGBE (1:1:1 ratio, vol/vol) to diet has the potential to improve milk yield and health status of cows under mild to moderate heat stress, denoting that it might be useful as an alternative anti-stressor in the diet of dairy cows under hot conditions.

## INTRODUCTION

By the end of the twenty-first century, it is likely that surface air temperatures will increase by 6°C across the Korean peninsula [1]. Consequently, there is likely to be an increase in the incidence of heat stress in livestock, which will influence their productivity. The temperature-humidity index (THI), which incorporates the effects of ambient temperature and relative humidity, is widely used as an indicator of the degree of heat stress [2]. Heat stress negatively affects a variety of animal production parameters (e.g., milk quality, feed intake, and milk yield). A study by Rhoads et al [3] showed that the dry matter intake (DMI) and milk yield are lower in heat-stressed cows than in cows that are kept in a thermoneutral environment. In addition to declines in feed intake and milk yield, significant decreases in milk quality have been reported under heat stress conditions [4]. Several management strategies for heat stress (e.g., provision of shade and cooling systems) have ameliorated some of the negative effects of thermal stress on animal productivity; however, productivity continues to decrease during heat events in summer, particularly in dairy cows.

One approach, dietary supplementation with a variety of natural resource extracts, is expected to alleviate the negative impact of heat events by improving health status and eventually contributing to increases in milk yield in dairy cows. Advances in supplementation with phytogenic or marine extracts as single components have improved the growth performance and health status of domestic animals by promoting appetite, enhancing feed digestibility, and improving immune responses and antioxidant capacity [5–7]. For instance, the compounds in garlic, such as S-allylcysteine, show great antioxidant potential by increasing the activity of several antioxidant enzymes [8,9]. A study by Kim et al [10] showed that the major components of phytonic oil extracted from pinecone are γ-terpinene, dl-limonene, 2-β-pinene, and isolongifolene, and that the components of pinecone oil have multiple biological properties including antioxidant and suppresion of cortisol. Brown seaweed is rich in a polysaccharide of alginic acid, and it been demonstrated that polysaccharide of fucoidan in brown seaweed shows antitumor, anticancer, and antioxidation effects [11,12]. However, information on supplementation with a mixture of phytogenic and marine extracts and its effects on cow performance and physiological traits is limited. Considering the adverse effects caused by heat events, we speculated that supplementation with the mixture of combined extracts would alleviate heat stress and improve animal performance and productivity. The present study was performed to evaluate the immunomodulatory potency of a mixture of phytogenic extracts (garlic and pinecone) and a marine extract (brown seaweed) using bovine mammary epithelial cells (MAC-T) *in vitro*. Based on the results of these experiments, we further examined the *in vivo* effects of a combination of phytogenic and marine extracts on milk production, milk quality, immune status, metabolic profiles, and behavior patterns of dairy cows experiencing heat stress during summer in the Republic of Korea.

## MATERIALS AND METHODS

The current study consisted of two experiments. In Experiment 1, a mixture of pinecone oil, garlic kernel, and brown seaweed midrib extracts (PGBE) was used to determine the optimal combination through analysis of radical scavenging activity using 2,2′-azino-bis (3-ethylberzothiazoline-6-sulphonic acid) (ABTS) and anti-inflammation using MAC-T to determine whether PGBE can be used as a feed additives in dairy cows. Based on the results from Experiment 1, we confirmed the impact of PGBE complex on milk production, milk composition, metabolic profiles, immune status, antioxidative parameters, and behavior patterns in dairy cows experiencing heat stress were evaluated in Experiment 2.

### Experiment 1: Screening for immunomodulatory potency of PGBE complex using MAC-T

#### Preparation of phytogenic and marine extracts and their mixtures

Extracts of pinecone oil, garlic kernel, and brown seaweed midrib were provided by the Phylus Corporation (Danyang, Korea). Each material—pinecone without pine nuts, garlic kernel, and brown seaweed midrib—was segmented into fragments of 2 to 3 cm in size, and 100 g of each was added into a 1 L flask. These were mixed with 500 mL distilled water for 1 to 2 min, followed by a distillation process at 100°C±3°C for 3 h. Each extract obtained using distillation was dehydrated with sodium sulfate for 24 h, and then collected, sealed, and stored at 4°C. To prepare the PGBE complex, the aforementioned extracts were mixed in the following combinations: 1:1:1, 2:1:1, 1:2:1, and 1:1:2 (vol/vol). All extractions were serially diluted with Dulbecco’s modified eagle’s medium (DMEM; Thermo Fisher Scientific Inc., Waltham, MA, USA) supplemented with 1% heat-inactivated fetal bovine serum (FBS; Gibco, Grand Island, NY, USA), filtered through a 0.22 μm membrane (MN sterilizer PES; Macherey-Nagel GmbH Co., Duren, Germany), and sealed and stored at 4°C until their use in the *in vitro* study.

#### Antioxidant activity using ABTS cation radical scavenging activity assay

The antioxidant capacity of the PGBE complex was investigated using ABTS cation radical scavenging activity assay, as described by Cai et al [13]. ABTS radical cations generated by reacting 7.2 millimolar (mM) ABTS and 2.6 mM potassium persulfate for 24 h in a dark room were diluted with phosphate-buffered saline (pH 7.4) so as to give 20% to 30% inhibition of the blank absorbance at 734 nm with a spectrophotometer (Biotek Instruments, Inc., Winooski, VT, USA). Subsequently, 10 μL of PGBE complex or L-ascorbic acid was added into 190 μL of ABTS solution to get a final PGBE concentration of 0.16% to 0.01% (vol/vol) or L-ascorbic acid (1.0, 0.1 and 0.01 mg/mL; Sigma-Aldrich, St. Louis, MO, USA) in a 96-well plate (Thermo Fisher, USA), and reacted for 10 min in a dark room. The optical density (OD) of the resulting mixture was measured at 734 nm, and radical scavenging activity was calculated using the following formula: 1 – (OD blank – OD sample)/OD blank.

#### Anti-inflammatory activity assessed using western blot analysis

MAC-T cells [14], immortalized bovine MAC-T, were cultured in DMEM (Thermo Fisher, USA) medium with 10% heat-inactivated FBS, 1% penicillin/streptomycin solution (Gibco, USA), 5 μg/mL bovine insulin (Sigma-Aldrich, USA), and 1 μg/mL progesterone (Sigma-Aldrich, USA) at 37°C in a humidified atmosphere of 95% air and 5% CO_2_. After reaching 90% confluence, cells were synchronized overnight in complete medium containing 1% heat-inactivated FBS before initiation of cell treatments. No mycoplasma was detected in the cultures.

Anti-inflammatory activity of PGBE was assessed using western blot analysis as described previously [15]. In brief, MAC-T cells (5×10^5^ cells/cm^2^) were grown to confluence and synchronized overnight in 6-well plates (Thermo Fisher, USA). Cells were pretreated at a concentration of 0.16% PGBE complex (1:1:1 ratio; vol/vol) for 6 h, and exposed to PBS or lipopolysaccharide *Escherichia coli* O111:B4 (LPS; Sigma-Aldrich, USA) at a concentration of 1 μg/mL for 12 h. For western blot analysis, 30 μg protein per lane from total extracts of each sample was separated using 10% sodium dodecyl sulfate-polyacrylamide gel electrophoresis (Mini-Protean Tetra Cell; Bio-Rad Laboratories, Inc., Hercules, CA, USA). Blots were exposed to X-ray film (Fujifilm Co., Tokyo, Japan) for 3 min and then scanned, and the band densities were quantified using ImageJ 1.43 software (National Institutes of Health. http://rsb.info.nih.gov/ij). Band densities were normalized to glyceraldehyde-3-phosphate dehydrogenase signals on the same membrane.

### Experiment 2: Effects of a supplemental mixture of PGBE on milk yield, milk composition, immune status, metabolic profiles, and behavior patterns in dairy cows experiencing heat stress

#### Cows, housing, experimental design, and treatments

The Animal Experimental Guidelines provided by the Animal Care and Use Committee of Konkuk University approved all procedures involving animals (KU16053), and the experiment was conducted (24 May to 03 July 2015) in Chugju at the University Farm Facility. A total of 40 lactating Holstein-Friesian cows were housed in a feedlot located in a roofed area with open sides and offered total mixed ration (TMR) and concentrated feed with free access to fresh water. Each pen (4 cows/pen) in the feedlot was equipped with automatic water troughs. Sawdust was used for bedding and refreshed once monthly. At the beginning of the study (24 May, day 0), cows (parity, 2.3±0.23; days in milk, 118±10.2 d; mean±standard deviation) averaging 37.4±1.28 kg/d milk yield were equipped with radio-frequency identification (RFID) ear tags (SensOor, Agis Automatisering BV, Harmelen, The Netherlands) and subsequently assigned to two different dietary groups. The total duration of the experiment was 40 d, during which the cows were fed a basal diet (control, n = 20) or a basal diet supplemented with 0.016% PGBE complex (PGBE, n = 20) at 09:00 h. The PGBE complex (pinecone oil, garlic kernel, and brown seaweed midrib extracts at ratio of 1:1:1 [vol/vol]) was premixed with the excipient (60% rice bran and 40% corn grit) at a 10% concentration and it was added to the TMR at a final concentration of 0.016% by top-dressing for *in vivo* study. Cows in the control group were fed the same amount of added the excipient (not included PGBE). The ingredients and chemical compositions of the diet are shown in Table 1. The basal diet was formulated to meet or exceed the nutrient requirements of NRC [16].

#### Temperature-humidity index

Ambient air temperature (AT, °C) and relative humidity (RH, %) inside the feedlot were recorded every 10 min daily using a self-made device (Korea Electronics Technology Institute, Seongnam, Korea) located at a height of 3 m from the ground. THI was calculated using the following equation [17]: THI = 0.8×AT+(RH×[AT–14.4]) +46.4.

#### Milk collection

Cows were milked twice daily (at 03:30 and 15:30 h), with individual milk yields recorded at each milking. Every 10 d, milk samples from two consecutive milking of individual cows were collected into a 50 mL tube containing bronopol-B2 preservative (D&F Control System Inc., Dublin, ON, Canada), pooled with same volume ratio, and kept at 4°C. Subsequently, the aliquot of milk was analyzed for fat, protein, lactose, somatic cell counts, solid-not fat, milk urea nitrogen, acetone, β-hydroxybutyrate, and casein-β, using a MilkoScan (CombiFoss FT+500 S/H, Hilleroed, Denmark).

#### Behavior patterns

Rumination and eating times of individual cows were monitored continuously using a 3-dimensional accelerometer attached to the RFID ear tag, which was placed in the proximal half of the ear between the two cartilage folds. Raw data transmitted from cows’ ear tags were analyzed via an online application (CowManager; Agis Automatisering BV, The Netherlands) provided by the manufacturer, which records time spent ruminating per hour, eating and ear surface temperature per day.

#### Blood collection

Blood collections were performed on day 0 and 40 of the experiment. Approximately 15 mL of duplicate blood samples from individual cows were collected from the jugular vein 4 h after feeding (at 09:00 h). One aliquot of blood sample was collected in serum separator tubes (Serum Clot Activator, Greiner Bio One GmbH, Kremsmunster, Austria), and the samples were allowed to clot for 30 min at room temperature and stored in the refrigerator overnight. Subsequently, serum was obtained by centrifugation at 1,300×*g* for 30 min at 4°C and used to determine the metabolic profile (blood urea nitrogen, albumin, glucose, non-esterified fatty acids, triglyceride, gamma-glutamyl transpeptidase, and creatine) and antioxidative parameters (total antioxidant capacity [TAC], erythrocyte glutathione [GSH], and thiobarbituric acid reactive substances) using a Toshiba Accute Biochemical Analyzer-TBA-40FR (Toshiba Medical Instruments, Tochigi-ken, Japan) and colorimetric procedures with commercially available enzyme-linked immunosorbent assay kits (Abcam plc., Cambridge, UK), respectively, according to the manufacturers’ instructions. Another aliquot of blood was collected into K_2_ ethylenediaminetetraacetic acid-containing vacutainer (BD Vacutainer, Franklin Lakes, NJ, USA) and immediately subjected to hematology using a VetScan HM2 Hematology System (Abaxis Inc., Union City, CA, USA) to evaluate the level of white blood cells, lymphocytes, monocytes, and granulocytes, according to the manufacturers’ instructions.

### Statistical analysis

Statistical analyses were conducted using the JMP 5.0 software package (SAS Institute Inc., Cary, NC, USA). A Tukey-Kramer honestly significant difference test was used to compare different dilution rates of PGBE complex against the vehicle alone and the radical scavenging activity of the PGBE complex compared to ascorbic acid in experiment 1 (Table 2). In addition, differences in the following variables compared with initial measurement (day 0) in experiment 2 were analyzed using the general linear model procedure of SAS and were determined using a Student’s *t*-test: differences in metabolic profiles, hematology, milk yield, milk composition, and anti-oxidative parameters (Tables 3, 4). The pen was the experimental unit. Milk yield and behavior patterns (Table 5) were analyzed using the MIXED model with repeated measures analyses. In the mixed model, the random effect was the animal into each pen and the least square means were compared using Tukey. Differences were considered statistically significant and tendency if the probability was less than 0.05 and between 0.05 and 0.1, respectively. Values obtained from experiment 1 and 2 are expressed as the mean with standard error of mean and mean with standard deviation, respectively.

## RESULTS AND DISCUSSION

### Experiment 1: Screening for immunomodulatory potency of the PGBE complex using MAC-T

The production of excessive reactive oxygen species, which causes oxidative stress and protein oxidation [18], is involved in the pathogenesis of many types of inflammatory disease. Various natural resources from land plants and marine algae with antioxidant effects have been used to treat inflammatory disease. We tested the antioxidant potency of the PGBE complex at various combination rates by estimating its ABTS cation radical scavenging activity (Table 2). All PGBE complexes at dilution rate of 0.16% showed strong antioxidant effects compared to ascorbic acid (1.0 mg/mL), but the radical scavenging activity of all PGBE complexes at dilution rates (0.08% to 0.01%) was weak and even disappeared. PGBE complex containing pinecone, garlic kernel, and brown seaweed midrib extracts (2:1:1; vol/vol) was comparable to the other combinations of PGBE (1:1:1 and 1:1:2; vol/vol), with the exception of PGBE combination 1:2:1; vol/vol (p = 0.0478). Consequently, 0.16% PGBE complex (1:1:1; vol/vol), as well as consideration of extract costs, was used to evaluate anti-inflammatory effects using MAC-T cells.

MAC-T cells, which are an immortalized epithelial cell line isolated from bovine mammary tissue, provide a useful *in vitro* model for bovine lactation because they retain a number of biochemical and morphological characteristics typical of bovine primary MAC-T *in vivo* [14]. Therefore, we suggest that examination of the anti-inflammatory potency of the PGBE complex using MAC-T cells in response to LPS might be useful to understand the similar inflammatory responses of heat stressed dairy cows. COX-2 expression is an indicator of inflammatory responses in a variety of cells, including MAC-T [19]. In order to identify the anti-inflammatory effects of PGBE complex (1:1:1, vol/vol), MAC-T cells were exposed to PGBE complex at concentrations of 0%, and 0.16% (vol/vol) for 6 h, followed by LPS treatment (0, 1 μg/mL) for 12 h. Western blot analysis showed that protein expression of COX-2 in MAC-T cells was elevated by LPS (1 μg/mL) as compared to non-LPS treatment (Supplementary Figure S1). Interestingly, we found that pretreatment with 0.16% PGBE complex (1:1:1; vol/vol) attenuated LPS-induced COX-2 expression in MAC-T cells.

Taken together, these *in vitro* trials suggest that the combination of PGBE at 1:1:1 (vol/vol) had the strongest antioxidative potency and anti-inflammatory effects by suppressing COX-2 expression in MAC-T cells under LPS stimulation.

### Experiment 2: Effects of a supplemental dietary mixture of PGBE on milk yield, milk composition, immune status, metabolic profiles, and behavior patterns in dairy cows experiencing heat stress

Based on the results of Experiment 1 and data previously described by Kim et al [10], we further examined the effects of supplemental dietary 0.016% PGBE complex (1:1:1, vol/vol) on milk yield, milk composition, immune status, metabolic profiles, and behavior patterns in dairy cows during heat events in the summer period compared to those of the basal diet to evaluate whether PBGE complex has potential to improve milk production.

Although there was no significant difference in milk composition and feed intake between the control and 0.016% PGBE-treated groups during the whole experimental period, the PBGE mixture showed increased milk yield for 40 days as compared to the control group (p<0.05) (Table 3). Comparing our observation, a study by Oh et al [20] showed that milk composition and blood chemistry were not affected by treatment with 2 g per cow of garlic extract for 9 d. DMI tended to be lower for the garlic treatment, which increased feed efficiency but slightly decreased milk yield. A study by Yang et al [21] investigated the effects of garlic (5 g per cow) and juniper berry (2 g per cow) essential oils on ruminal fermentation and on the site and extent of digestion in lactating cows, and reported that milk yield did not change. A prior study showed that incorporation of 0.016% pinecone oil into basal diet did not have a large influence on milk yield or metabolic and hematological parameters in lactating Holstein cows [10]. A study by Hong et al [5] found that feeding dietary brown seaweed byproducts at 2% and 4% for 12 months did not affect DMI, milk yield, or milk composition in Holstein cows. In addition, Benchaar et al [22] observed no changes in milk yield or milk composition in cows supplemented with 2 g per day or 750 mg per day of a commercial mixture of essential oil compounds, reporting that milk protein and milk lactose content, as well as their yields, were not affected by the treatment. Similarly, milk efficiency, presented either as kg of milk or as fat-corrected milk per kg of DMI, did not differ among the groups. Although there was no observed improvement in milk composition following supplementation with the combined mixture of phytogenic extracts (garlic and pinecone) and marine extract (brown seaweed) in the current study, we suggest that supplementation with 0.016% PGBE at 1:1:1 (vol/vol) in basal diet was effective for improving the milk yield of lactating cows.

A study by Yun et al [9] showed that a garlic bulb consists of the organosulfur compounds alliin, γ-glutamyl-S-allylcysteine, S-methyl cysteine sulfoxide, S-trans-1-propenylcysteine sulfoxide, S-2-carboxypropylglutathione, and S-allylcysteine. As aforementioned, the compounds in garlic, such as S-allylcysteine, show great antioxidant potential by increasing the activity of several antioxidant enzymes, such as GSH reductase, superoxide dismutase, and g-glutamate cysteine ligase [8,9]. A study by Kim et al [10] showed that the major components of phytonic essential oil extracted from pinecone are γ-terpinene, dl-limonene, 2-β-pinene, and isolongifolene, and that the components of pinecone oil have multiple biological properties including antioxidant and suppression of cortisol. Brown seaweed is rich in a polysaccharide of alginic acid, and it been demonstrated that polysaccharide of fucoidan in brown seaweed shows anticoagulation, antitumor, anticancer, and antioxidation effects [11,12]. In the blood traits of cows, we did not observe a significant difference in antioxidant ability (TAC and GSH) (Table 4). However, cows in the control group demonstrated significantly increased thiobarbituric acid reactive substances levels for 40 days (p<0.05), but cows supplemented with 0.016% PGBE in basal diet for 40 days showed tendency of an elevation of TAC (p = 0.0751) and GSH concentration (p = 0.0894) compared to those on day 0. Combined with the results in Table 2, we suggest that 0.016% PGBE complex can scavenge free radicals, causing an antioxidant effect in lactating cows. There were no significant differences in metabolic (blood urea nitrogen, albumin, glucose, non-esterified fatty acid, triglyceride, gamma-glutamyl transpeptidase, and creatine) or hematological (white blood cell, lymphocyte, monocyte, and granulocyte) parameters between the control and 0.016% PGBE-treated groups.

The THI is a measure that accounts for the combined effects of environmental temperature and relative humidity on cattle to assess the risk of heat stress and prevent major effects. Heat stress causes changes in homeostasis and has been quantified by the measurement of milk production and physiological variables such as body temperature, respiratory rate, and behaviors of cows [23]. According to a study by Gernand et al [24], milk production is not affected by heat stress when mean THI values are under 68. However, milk production and feed intake begin to decline when beyond THI 68 [24,25]. During the experimental period of 40 days, animals were subjected to mild to moderate stress conditions (temperature, 25.4°C±2.58°C; humidity, 58.8%±14.15% [THI = 73.0±3.05]) in the current study (Supplementary Figure S2). Considering the conditions using THI values, we further evaluated the data to determine the effects on milk yield and behavior patterns (rumination, eating, and ear surface temperature) of cows based on THI values such as stress threshold (temperature, 19.2°C±0.55°C; humidity, 78.8%± 4.53% [THI = 65.6±1.05]) and mild to moderate stress conditions (temperature, 26.1°C±0.33°C; humidity, 57.0%±2.29% [THI = 73.8±0.38]) (Table 5). Milk yield between groups was not affected by THI, dietary supplementation, or THI interaction. For milk yield, however, cows experiencing mild to moderate stress (THI = 73.8) in the control group demonstrated tendency of a reduction of milk yield (p = 0.0781) compared with cows under the stress threshold (THI = 65.6). By contrast, when the THI value exceeded 72 (average THI = 73.8), the milk yield in 0.016% PGBE-treated group was greater as compared to that in the control group (p<0.05). Moreover, we observed a tendency of increase in milk yield of cows in the PGBE-treated group as compared to the control group under stress threshold conditions (p = 0.0897), suggesting that incorporation of 0.016% PGBE into the diet improved the milk yield as compared to non-treatment (control) under the stress threshold and even in mild to moderate stress conditions. Rumination is reduced in cows experiencing heat stress [26]. A study by Moallem et al [26] reported that the primary negative effect of THI is depression of rumination time, which subsequently leads to a reduction in DMI, followed by decreased milk yield. In the current study, the mild to moderate stress (THI = 73.8) led to a lower percentage of rumination time of cows in the control group compared to the initial period (control = −2.2%). There was no difference in the percentage of rumination time of cows in the PGBE-treated groups according to THI value. When THI was 73.8, the rumination time of cows in the 0.016% PGBE-treated group showed a tendency of an elevation compared to that in the control group (control = −2.2% vs 0.016% PGBE = 0.8%, p = 0.0914). In the increment, all cows showed a decline in eating and ear surface temperature as THI increased compared to the initial period. Cow behavior studies have shown that cows suffering heat stress eat more frequent, smaller meals and, to increase the surface area available for dissipating body temperature, stand for a longer time and pant as ambient temperature increases [27]. Chewing and rumination might be impaired by panting in heat-stressed cows, particularly during the daytime. Despite a reduction in rumination in the control group, increased rumination of cows in the PGBE-treated group during the mild to moderate stress period was observed in the current study. We can hypothesize that cows supplemented with 0.016% PGBE might prevent deterioration of depression of rumination time, which subsequently leads to a reduction in DMI and milk yield, when THI was increased

In conclusion, the lactating dairy cows used in this study suffered mild to moderate heat stress throughout the experimental period (THI = 73.0). The milk production of cows experiencing heat stress in this trial showed an increase with supplementation of 0.016% PGBE complex in the diet. Besides showing antioxidant potency and anti-inflammatory ability, the PGBE complex (1:1:1 ratio, vol/vol), as shown in the aforementioned results in Table 2 and Supplementary Figure S1, prevented a decrease in the milk yield of cows during some periods of the trial (THI = 73.8). In particular, rumination time was negatively affected by hot conditions, but cows supplemented with PGBE complex exhibited increased rumination compared to the control. Our results suggest that incorporation of a combined mixture of PGBE to diet has the potential to improve milk yield and health status of cows under mild to moderate heat stress. Therefore, 0.016% PGBE (1:1:1 ratio, vol/vol) might be useful as an alternative anti-stressor in the diet of lactating dairy cows under hot conditions.

## Supplementary Data





## Figures and Tables

**Table 1:** Ingredient and chemical composition of the experimental diets used in Experiment 2

Items	TMR	Concentrates	Excipients
Diet ingredient, % of DM			
Canola seed meal	-	9.00	-
Coconut kernel meal	-	6.00	-
Corn	-	20.00	-
Corn gluten feed	5.00	13.60	-
Dicalcium phospate	-	0.20	-
Limestone	-	1.50	-
MgO	-	0.20	-
Molasses	1.50	6.00	-
Parm kernel meal	1.00	14.20	-
Salt	-	0.50	-
Sesame seed meal	-	2.00	-
Sodium bicarbonate	-	0.40	-
Soybean meal	2.00	10.00	-
Tallow	-	2.00	-
Tapioca	-	3.00	-
Vitamin mineral premix1)	-	0.40	-
Wheat middling	-	11.00	-
Canola seed meal	-	9.00	-
Alfalfa hay	6.50	-	-
Beef pulp	2.50	-	-
Concentrate	18.00	-	-
Corn flake	9.00	-	-
Cotton seed	5.00	-	-
Klein grass hay	5.00	-	-
Oaten hay	13.00	-	-
Timothy hay	2.50	-	-
Rice bran	-	-	60
Corn grit	-	-	40
DM of feed (kg/d)	25.05	2.65	0.045
Chemical composition, kg/d of DM (unless otherwise noted)			
Crude protein	3.14	0.52	0.005
Ether extract	0.72	0.13	0.004
Crude fiber	3.64	0.23	0.002
Crude ash	1.22	0.22	0.002
NDF	7.09	0.68	-
ADF	3.52	0.29	-
Ca	0.16	0.03	<0.000
P	0.09	0.02	<0.000
NE_L_ (Mcal/kg of DM)2)	2.15	2.10	2.32

Cows were fed basal diet (control) or basal diet supplemented with 0.016% PGBE complex (PGBE; DM basis) for 40 d. The basal diet was also used for feeding during the pretrial period.

TMR, total mixed ration; DM, dry matter; NDF, neutral detergent fiber; ADF, acid detergent fiber; NEL, net energy of lactation.

1) Vitamin mineral premix: 1,000,000 IU Vit. A, 100,000 IU Vit. D_3_, 25,000 mg Vit. E, 150 mg I, 150 mg Co, 2,500 mg Cu, 6,250 mg Fe, 16,000 mg Mn, 10,000 mg Zn, 150 mg Se.

2) NE_L_: Mcal/kg of DM was estimated based on NRC [16].

**Table 2:** ABTS cation radical scavenging activity of PGBE complex

PGBE ratio1)	Dilution rate2)					Ascorbic acid (mg/mL)		
	
	0.16	0.08	0.04	0.02	0.01	1.0	0.1	0.01
1:1:1	84±11.3^b^,^AB^	6±2.8^d^	3±9.6^d^	0.4±1.0^d^	0	96±0.7^a^	54±5.0^c^	10±13.3^d^
2:1:1	87±4.2^a^,^A^	8±5.9^c^	9±4.6^c^	0.4±5.0^c^	0	96±0.7^a^	54±5.0^b^	10±13.3^c^
1:2:1	69±4.7^b^,^B^	0	0	0	0	96±0.7^a^	54±5.0^b^	10±13.3^c^
1:1:2	85±0.8^b^,^AB^	0	0	0	0	96±0.7^a^	54±5.0^c^	10±13.3^d^

Each value represents the means and standard errors of four replicates.

ABTS, 2,2′-azino-bis (3-ethylberzothiazoline-6-sulphonic acid); PGBE, a mixture of pinecone oil, garlic kernel, and brown seaweed midrib extracts.

1) PGBE ratio represented as a combination ratio of pinecone oil, garlic kernel, and brown seaweed midrib extracts (vol/vol).

2) Dilution represented as a ratio of extract and complete medium (%, vol/vol).

a–c Values that differed among diluted groups in a row are significantly different at p<0.05 by a Tukey test.

A–C Differing values among mixture rates in a column are significantly different at p<0.05 by the Tukey test.

**Table 3:** Effects of PGBE complex on milk yield and composition in dairy cows

Items	Control		PGBE1)	
	
	D 0	D 1–40	D 0	D 1–40
Milk yield (kg/d)	37.9±1.85	37.6±0.12	36.8±1.85	39.0±0.03*
Feed intake (kg of DM/d)	27.5±2.41	27.7±1.43	27.7±1.15	27.6±2.05
TMR	25.0±1.21	25.1±0.95	25.0±0.66	25.1±1.34
Concentrate	2.6±0.12	2.6±0.42	2.6±0.88	2.6±0.57
Milk composition				
Fat (%)	4.07±0.513	4.00±0.606	4.15±0.452	4.04±0.659
Protein (%)	3.19±0.201	3.16±0.217	3.23±0.280	3.24±0.286
Lactose (%)	4.78±0.174	4.78±0.187	4.80±0.105	4.84±0.135
SNF (%)	8.54±0.244	8.59±0.271	8.57±0.304	8.70±0.292
SCC (×1,000/mL)	150.5±215.43	158.5±433.76	83.1±134.52	104.3±193.9
MUN (mg/dL)	18.8±2.80	17.5±2.900	18.9±3.36	17.8±2.65
Acetone (mM)	0.08±0.038	0.10±0.048	0.08±0.024	0.09±0.041
BHBA (mM)	0.02±0.017	0.02±0.019	0.02±0.020	0.02±0.017
Casein-β (%)	2.34±0.167	2.35±0.185	2.38±0.225	2.42±0.234

Values represent the means plus standard deviation of 20 individual dairy cows.

PGBE, a mixture of pinecone oil, garlic kernel, and brown seaweed midrib extracts; TMR, total mixed ration; SNF, solids-not fat; SCC, somatic cell count; MUN, milk urea nitrogen; BHBA, β-hydroxybutyrate.

1) PGBE: mixture of pinecone oil, garlic kernel, and brown seaweed midrib extracts at ratio of 1:1:1 (vol/vol).

* Indicates a significant difference compared to the zero day at p<0.05.

**Table 4:** Effects of PGBE complex on blood traits in dairy cows

Traits	Control		PGBE1)	
	
	D 0	D 40	D 0	D 40
Metabolic parameters				
BUN (mg/dL)	12.4±1.44	11.5±0.75	12.3±0.54	11.8±0.47
Albumin (g/dL)	3.3±0.21	3.2±0.49	3.5±0.11	3.7±0.10
Glucose (mg/dL)	54.0±4.62	54.7±4.23	53.4±4.12	53.2±4.34
NEFA (mmol/L)	269.7±14.30	236.1±45.86	296.7±21.30	257.7±35.58
Triglyceride (mg/dL)	14.6±1.02	15.5±4.13	13.6±5.02	14.7±4.16
γ-GTP (IU/L)	32.6±9.08	27.6±12.85	31.6±9.08	26.0±10.36
Creatine (mg/dL)	0.84±0.032	0.74±0.324	0.74±0.052	0.75±0.027
Hematology				
WBC, 4–12 K/μL	15.43±1.285	15.75±1.005	13.21±1.202	11.48±1.039
Lymphocyte, 2.5–7.5 K/μL	9.50±1.145	10.35±1.081	8.01±0.915	6.15±0.800
Monocyte, 0–0.84 K/μL	1.27±0.168	0.89±0.151	1.03±0.146	0.79±0.114
Granulocyte, 0.6–6.7 K/μL	4.66±0.307	5.10±0.661	3.98±0.350	4.36±0.386
Antioxidant parameters				
TAC (μM)	140.4±3.64	144.0±3.78	138.7±3.78	145.2±2.92
GSH (μM)	88.2±9.75	82.2±9.48	75.3±2.24	85.5±8.75
TBARS (μM)	0.78±0.173	1.01±0.085*	0.65±0.042	0.94±0.283

Values represent the means and standard deviation of 20 individual dairy cows.

PGBE, a mixture of pinecone oil, garlic kernel, and brown seaweed midrib extracts; BUN, blood urea nitrogen; NEFA, non-esterified fatty acids; γ-GTP, gamma-glutamyl transpeptidase; WBC, white blood cells; TAC, total antioxidant capacity; GSH, glutathione; TBARS, thiobarbituric acid reactive substances.

1) PGBE: mixture of pinecone oil, garlic kernel, and brown seaweed midrib extracts at ratio of 1:1:1 (vol/vol).

* Indicates a significant difference compared to value at the initial day (D 0) at p<0.05.

**Table 5:** Effects of PGBE complex on milk yield, and behavior patterns in dairy cows based on the average temperature-humidity index

Items	Control		PGBE1)		p value		
		
	THI_avg_				Diet (D)	THI (T)	D×T

	65.6	73.8	65.6	73.8			
Milk yield (kg/d)							
Initial	37.9±1.85	-	36.8±1.85	-			
Experiment	38.2±1.00	37.5±1.70	39.0±0.99	38.9±0.27			
Increment2)	+0.4±0.46^ab^	−0.4±0.46^b^	+2.3±0.88^a^	+2.2±0.97^a^	0.0024	0.5497	0.6356
Behavior patterns							
Rumination (%)	-	-	-	-			
Initial	40.8±0.97	-	38.5±1.39	-			
Experiment	39.5±0.01	38.6±0.73	40.9±1.21	39.3±0.90			
Increment	−1.3±0.74	−2.2±0.64	+2.4±1.40	+0.8±1.09	0.7619	0.8577	0.7207
Eating (%)							
Initial	24.4±1.59	-	24.7±1.83	-			
Experiment	24.0±1.60	23.1±1.46	22.9±1.83	23.2±1.57			
Increment	−0.5±0.82	−1.3±0.74	−1.8±1.13	−1.6±0.93	0.3837	0.7538	0.5243
Ear surface temperature (°C)							
Initial	31.7±0.21	-	31.4±0.25	-			
Experiment	29.2±0.33	32.8±0.27	29.0±0.30	32.2±0.16			
Increment	−2.5±0.24	+1.0±0.23	−3.6±1.59	−0.9±1.71	0.3202	<0.0001	0.7734

Values represent the means and standard deviation of 20 individual dairy cows.

PGBE, a mixture of pinecone oil, garlic kernel, and brown seaweed midrib extracts; THI_avg_, average temperature-humidity index.

1) PGBE: mixture of pinecone oil, garlic kernel, and brown seaweed midrib extracts at ratio of 1:1:1 (vol/vol).

2) Increment: values of experiment vs initial.

a,b means in the same row with different superscripts differ (p<0.05).
