# Supplementary material for: Dietary supplementation with combined extracts from garlic (Allium sativum), brown seaweed (Undaria pinnatifida), and pinecone (Pinus koraiensis) improves milk production in Holstein cows under heat stress conditions
Source: Asian-Australas J Anim Sci. 2019 Nov 12;33(1):111–9. doi: 10.5713/ajas.19.0536 (PMC6946988; doi:10.5713/ajas.19.0536)
Supplement: Supplementary file 1 [file ajas-19-0536-suppl1.pdf]

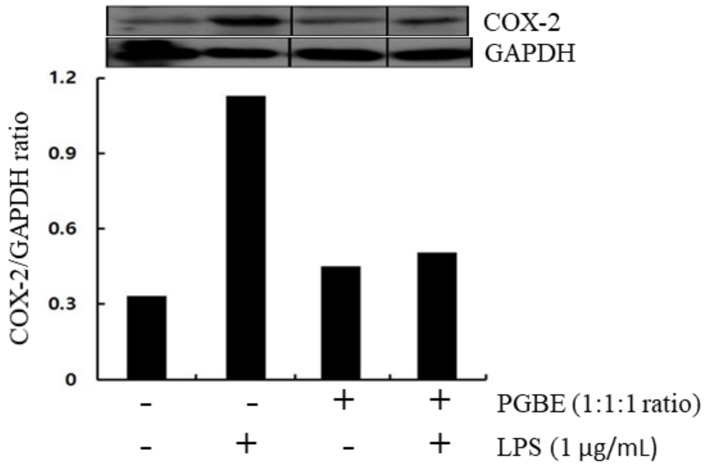

Suppl. 1. Cox-2 expression of bovine mammary epithelial cells during *in vitro* stimulation without or with PGBE complex followed by lipopolysaccharide (LPS; 1 μL/mL) induction. PGBE contained a mixture of pinecone oil, garlic, and brown seaweed extracts at ratio of 1:1:1 (vol/vol).
