# Supplementary material for: Dietary supplementation with combined extracts from garlic (Allium sativum), brown seaweed (Undaria pinnatifida), and pinecone (Pinus koraiensis) improves milk production in Holstein cows under heat stress conditions
Source: Asian-Australas J Anim Sci. 2019 Nov 12;33(1):111–9. doi: 10.5713/ajas.19.0536 (PMC6946988; doi:10.5713/ajas.19.0536)
Supplement: Supplementary file 2 [file ajas-19-0536-suppl2.pdf]

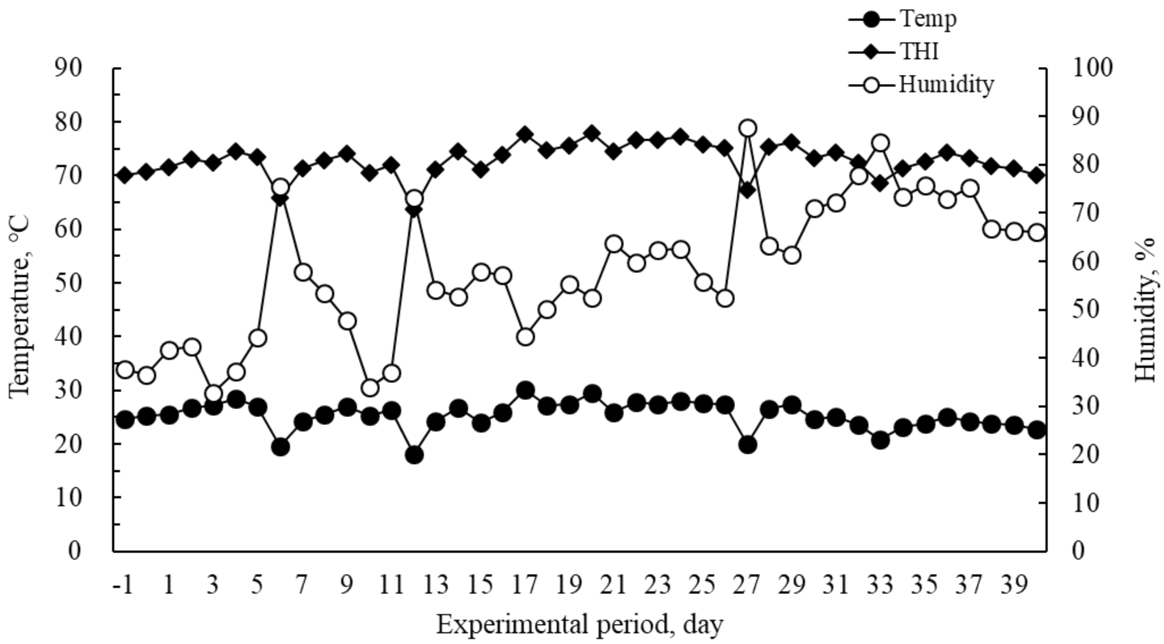

Suppl. 2. Changes in the temperature-humidity index (THI) formulated from temperature and humidity during the experimental period. THI was calculated as daily mean THI based on ambient air temperature (AT; °C) and relative humidity (RH; %) using following equation [17]: THI = 0.8 × AT + [RH × (AT– 14.4)] + 46.4.
